# Connecting extracellular metabolomic measurements to intracellular flux states in yeast

**DOI:** 10.1186/1752-0509-3-37

**Published:** 2009-03-25

**Authors:** Monica L Mo, Bernhard Ø Palsson, Markus J Herrgård

**Affiliations:** 1 Department of Bioengineering, University of California, San Diego, La Jolla, CA 92093, USA; 2 Current address: Synthetic Genomics, Inc, 11149 N Torrey Pines Rd, La Jolla, CA 92037, USA

## Abstract

**Background:**

Metabolomics has emerged as a powerful tool in the quantitative identification of physiological and disease-induced biological states. Extracellular metabolome or metabolic profiling data, in particular, can provide an insightful view of intracellular physiological states in a noninvasive manner.

**Results:**

We used an updated genome-scale metabolic network model of Saccharomyces cerevisiae, *i*MM904, to investigate how changes in the extracellular metabolome can be used to study systemic changes in intracellular metabolic states. The *i*MM904 metabolic network was reconstructed based on an existing genome-scale network, *i*ND750, and includes 904 genes and 1,412 reactions. The network model was first validated by comparing 2,888 in silico single-gene deletion strain growth phenotype predictions to published experimental data. Extracellular metabolome data measured in response to environmental and genetic perturbations of ammonium assimilation pathways was then integrated with the *i*MM904 network in the form of relative overflow secretion constraints and a flux sampling approach was used to characterize candidate flux distributions allowed by these constraints. Predicted intracellular flux changes were consistent with published measurements on intracellular metabolite levels and fluxes. Patterns of predicted intracellular flux changes could also be used to correctly identify the regions of the metabolic network that were perturbed.

**Conclusion:**

Our results indicate that integrating quantitative extracellular metabolomic profiles in a constraint-based framework enables inferring changes in intracellular metabolic flux states. Similar methods could potentially be applied towards analyzing biofluid metabolome variations related to human physiological and disease states.

## Background

"Omics" technologies are rapidly generating high amounts of data at varying levels of biological detail. In addition, there is a rapidly growing literature and accompanying databases that compile this information. This has provided the basis for the assembly of genome-scale metabolic networks for various microbial and eukaryotic organisms [1-11]. These network reconstructions serve as manually curated knowledge bases of biological information as well as mathematical representations of biochemical components and interactions specific to each organism.

A genome-scale network reconstruction is a structured collection of genes, proteins, biochemical reactions, and metabolites determined to exist and operate within a particular organism. This network can be converted into a predictive model that enables *in silico *simulations of allowable network states based on governing physico-chemical and genetic constraints [12,13]. A wide range of constraint-based methods have been developed and applied in order to analyze network metabolic capabilities under different environmental and genetic conditions [13]. These methods have been extensively used to study genome-scale metabolic networks and have successfully predicted, for example, optimal metabolic states, gene deletion lethality, and adaptive evolutionary endpoints [14-16]. Most of these applications utilize optimization-based methods such as flux balance analysis (FBA) to explore the metabolic flux space. However, the behavior of genome-scale metabolic networks can also be studied using unbiased approaches such as uniform random sampling of steady-state flux distributions [17]. Instead of identifying a single optimal flux distribution based on a given optimization criterion (e.g. biomass production), these methods allow statistical analysis of a large range of possible alternative flux solutions determined by constraints imposed on the network. Sampling methods have been previously used to study global organization of *E. coli *metabolism [18] as well as to identify candidate disease states in the cardiomyocyte mitochondria [19].

Network reconstructions provide a structured framework to systematically integrate and analyze disparate datasets including transcriptomic, proteomic, metabolomic, and fluxomic data. Metabolomic data is one of the more relevant data types for this type of analysis as network reconstructions define the biochemical links between metabolites, and recent advancements in analytical technologies have allowed increasingly comprehensive intracellular and extracellular metabolite level measurements [20,21]. The metabolome is the set of metabolites present under a given physiological condition at a particular time and is the culminating phenotype resulting from various "upstream" control mechanisms of metabolic processes. Of particular interest to this present study are the quantitative profiles of metabolites that are secreted into the extracellular environment by cells under different conditions. Recent advances in profiling the extracellular metabolome (EM) have allowed obtaining insightful biological information on cellular metabolism without disrupting the cell itself. This information can be obtained through various analytical detection, identification, and quantization techniques for a variety of systems ranging from unicellular model organisms to human biofluids [20-23].

Metabolite secretion by a cell reflects its internal metabolic state, and its composition varies in response to genetic or experimental perturbations due to changes in intracellular pathway activities involved in the production and utilization of extracellular metabolites [21]. Variations in metabolic fluxes can be reflected in EM changes which can, in turn, provide insight into the intracellular pathway activities related to metabolite secretion. The extracellular metabolomic approach has already shown promise in a variety of applications, including capturing detailed metabolite biomarker variations related to disease and drug-induced states and characterizing gene functions in yeast [24-27]. However, interpreting changes in the extracellular metabolome can be challenging due to the indirect relationship between the proximal cause of the change (e.g. a mutation) and metabolite secretion.

Since metabolic networks describe mechanistic, biochemical links between metabolites, integration of such data can allow a systematic approach to identifying altered pathways linked to observed quantitative changes in secretion profiles. Measured secretion rates of major byproduct metabolites can be applied as additional exchange flux constraints that define observed metabolic behavior. For example, a recent study integrating small-scale EM data with a genome-scale yeast model correctly predicted oxygen consumption and ethanol production capacities in mutant strains with respiratory deficiencies [28]. The respiratory deficient mutant study used high accuracy measurements for a small number of major byproduct secretion rates together with an optimization-based method that are well suited for such data. Here, we expand the application range of the model-based method used in [28] to extracellular metabolome profiles, which represent a temporal snapshot of the relative abundance for a larger number of secreted metabolites. Our approach is complementary to statistical (i.e. "top-down") approaches to metabolome analysis [29] and can potentially be used in applications such as biofluid-based diagnostics or large-scale characterization of mutants strains using metabolite profiles.

**Figure 1:**
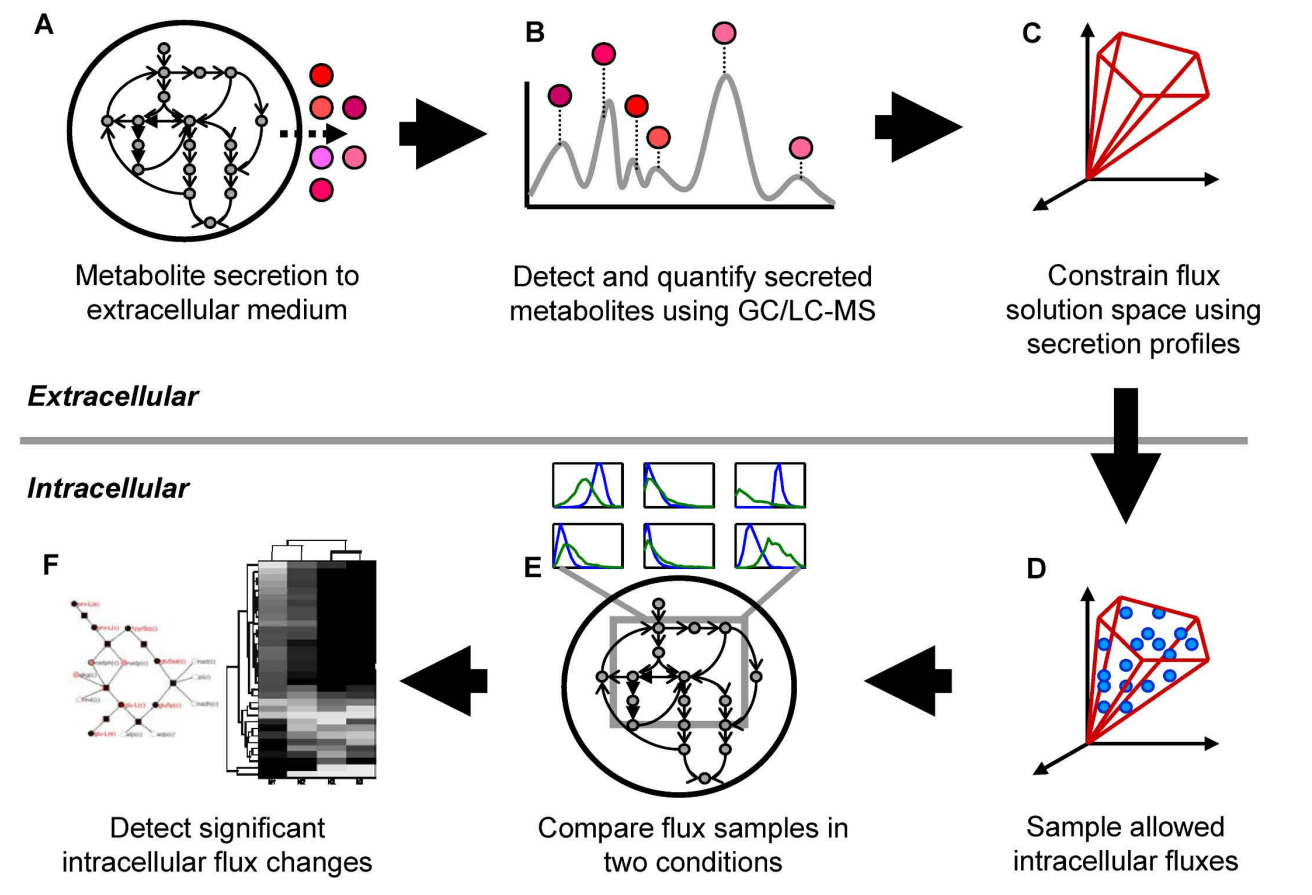
**Schematic illustrating the integration of exometabolomic (EM) data with the constraint-based framework**. (A) Cells are subjected to genetic and/or environmental perturbations to secrete metabolite patterns unique to that condition. (B) EM is detected, identified, and quantified. (C) EM data is integrated as required secretion flux constraints to define allowable solution space. (D) Random sampling of solution space yields the range of feasible flux distributions for intracellular reactions. (E) Sampled fluxes were compared to sampled fluxes of another condition to determine which metabolic regions were altered between the two conditions (see Figure 2). (F) Significantly altered metabolic regions were identified.

**Figure 2:**
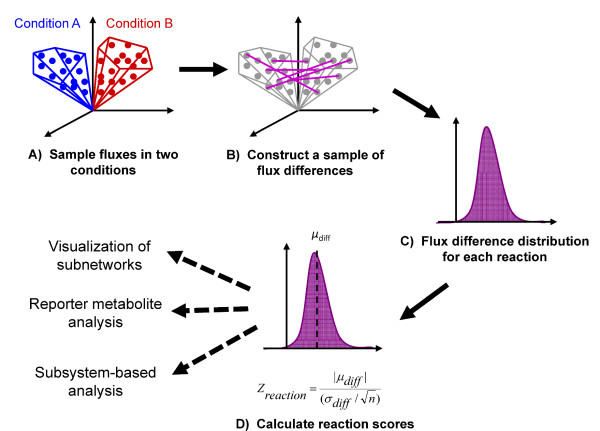
**Schematic of sampling and scoring analysis to determine intracellular flux changes**. (A) Reaction fluxes are sampled for two conditions. (B & C) Sample of flux differences is calculated by selecting random flux values from each condition to obtain a distribution of flux differences for each reaction. (D) Standardized reaction *Z*-scores are determined, which represent how far the sampled flux differences deviates from a zero flux change. Reaction scores can be used in visualizing perturbation subnetworks and analyzing reporter metabolites and subsystems.

In this study, we implemented a constraint-based sampling approach on an updated genome-scale network of yeast metabolism to systematically determine how EM level variations are linked to global changes in intracellular metabolic flux states. By using a sampling-based network approach and statistical methods (Figure 1), EM changes were linked to systemic intracellular flux perturbations in an unbiased manner without relying on defining single optimal flux distributions as was used in the previously mentioned study [28]. The inferred perturbations in intracellular reaction fluxes were further analyzed using reporter metabolite and subsystem (i.e., metabolic pathway) approaches [30] in order to identify dominant metabolic features that are collectively perturbed (Figure 2). The sampling-based approach also has the additional benefit of being less sensitive to inaccuracies in metabolite secretion profiles than optimization-based methods and thus can more readily be used in settings such as biofluid metabolome analysis.

This study was divided into two parts and describes: (i) the reconstruction and validation of an expanded *S. cerevisiae *metabolic network, *i*MM904; and (ii) the systematic inference of intracellular metabolic states from two yeast EM data sets using a constraint-based sampling approach. The first EM data set compares wild type yeast to the *gdh1/GDH2 *(glutamate dehydrogenase) strain [31], which indicated good agreement between predicted metabolic changes of intracellular metabolite levels and fluxes [31,32]. The second EM data set focused on secreted amino acid measurements from a separate study of yeast cultured in different ammonium and potassium concentrations [33]. We analyzed the EM data to gain further insight into perturbed ammonium assimilation processes as well as metabolic states relating potassium limitation and ammonium excess conditions to one another. The model-based analysis of both separately published extracellular metabolome datasets suggests a relationship between glutamate, threonine and folate metabolism, which are collectively perturbed when ammonium assimilation processes are broadly disrupted either by environmental (excess ammonia) or genetic (gene deletion/overexpression) perturbations. The methods herein present an approach to interpreting extracellular metabolome data and associating these measured secreted metabolite variations to changes in intracellular metabolic network states.

## Methods

### Metabolic network reconstruction

The previously reconstructed *i*ND750, a fully compartmentalized and elementally-balanced *S. cerevisiae *metabolic network, was used as the basis for reconstructing the *i*MM904 network [2]. The network was further expanded to include additional genes and reactions based on genomic, biochemical, and physiological information [see Additional file 1]. The details of existing reactions (substrate and cofactor specificity, reaction reversibility, and compartmentalization) in the *i*ND750 network were also re-evaluated to update the model based on existing literature. The *i*MM904 network was reconstructed using the SimPheny^® ^modeling software (Genomatica Inc, San Diego, CA). Existing gene-protein-reaction (GPR) associations from *i*ND750 were also reviewed and several were modified to include additional genes and proteins. GPR associations are Boolean representations of the logical relationship between ORFs and their corresponding transcripts, proteins, and reactions to enable mapping of genes to their respective functions. The included model text files [see Additional file 2] are compatible for computation with the COBRA toolbox [13].

### Conversion of the network to a predictive model

The network reconstruction was converted to a constraint-based model using established procedures [13]. Network reactions and metabolites were assembled into a stoichiometric matrix **S **containing the stoichiometric coefficients of the reactions in the network. The steady-state solution space containing possible flux distributions is determined by calculating the null space of **S**: **S **^. ^***v ***= 0, where ***v ***is the reaction flux vector. Minimal media conditions were set through constraints on exchange fluxes corresponding to the experimental measured substrate uptake rates. All the model-based calculations were done using the Matlab COBRA Toolbox [13] utilizing the glpk or Tomlab/CPLEX (Tomopt, Inc.) optimization solvers.

### Chemostat growth simulations

The *i*MM904 model was initially validated by simulating wild type yeast growth in aerobic and anaerobic carbon-limited chemostat conditions and comparing the simulation results to published experimental data on substrate uptake and byproduct secretion in these conditions [34]. The study was performed following the approach taken to validate the *i*FF708 model in a previous study [35]. The predicted glucose uptake rates were determined by setting the *in silico *growth rate to the measured dilution rate, which are equivalent under continuous culture growth, and minimizing the glucose uptake rate. The accuracy of *in silico *predictions of substrate uptake and byproduct secretion by the *i*MM904 model was similar to the accuracy obtained using the *i*FF708 model and results are shown in Figure S1 [see Additional file 3].

### Genome-scale gene deletion phenotype predictions

The *i*MM904 network was further validated by performing genome-scale gene lethality computations following established procedures to determine growth phenotypes under minimal medium conditions and compared to published data. A modified version of the biomass function used in previous *i*ND750 studies was set as the objective to be maximized and gene deletions were simulated by setting the flux through the corresponding reaction(s) to zero. The biomass function was based on the experimentally measured composition of major cellular constituents during exponential growth of yeast cells and was reformulated to include trace amounts of additional cofactors and metabolites with the assumed fractional contribution of 10^-6^. These additional biomass compounds were included according to the biomass formulation used in the *i*LL672 study to improve lethality predictions through the inclusion of additional essential biomass components [3]. The model was constrained by limiting the carbon source uptake to 10 mmol/h/gDW and oxygen uptake to 2 mmol/h/gDW. Ammonia, phosphate, and sulfate were assumed to be non-limiting. The experimental phenotyping data was obtained using strains that were auxotrophic for methionine, leucine, histidine, and uracil. These auxotrophies were simulated by deleting the appropriate genes from the model and supplementing the *in silico *strain with the appropriate supplements at non-limiting, but low levels. Furthermore, trace amounts of essential nutrients that are present in the experimental minimal media formulation (4-aminobenzoate, biotin, inositol, nicotinate, panthothenate, thiamin) were supplied in the simulations [3].

Three distinct methods to simulate the outcome of gene deletions were utilized: Flux-balance analysis (FBA) [36-38], Minimization of Metabolic Adjustment (MoMA) [39], and a linear version of MoMA (linearMoMA). In the linearMoMA method, minimization of the quadratic objective function of the original MoMA algorithm was replaced by minimization of the corresponding 1-norm objective function (i.e. sum of the absolute values of the differences of wild type FBA solution and the knockout strain flux solution). The computed results were then compared to growth phenotype data (viable/lethal) from a previously published experimental gene deletion study [3].

The comparison between experimental and *in silico *deletion phenotypes involved choosing a threshold for the predicted relative growth rate of a deletion strain that is considered to be viable. We used standard ROC curve analysis to assess the accuracy of different prediction methods and models across the full range of the viability threshold parameter, with results shown in Figure S2 [see Additional file 3]. The ROC curve plots the true viable rate against the false viable rate thus allowing comparison of different models and methods without requiring arbitrarily choosing this parameter *a priori *[40]. The optimal prediction performance corresponds to the point closest to the top left corner of the ROC plot (i.e. 100% true viable rate, 0% false viable rate). The values reported in Table 1 correspond to selecting the optimal viability threshold based on this criterion. We summarized the overall prediction accuracy of a model and method using the Matthews Correlation Coefficient (MCC) [40]. The MCC ranges from -1 (all predictions incorrect) to +1 (all predictions correct) and is suitable for summarizing overall prediction performance in our case where there are substantially more viable than lethal gene deletions. ROC plots were produced in Matlab (Mathworks, Inc.).

**Table 1:** Comparison of *i*MM904 and *i*LL672 gene deletion predictions and experimental data under minimal media conditions.

**Media**	**Model**	**Method**	**True viable**	**False viable**	**False lethal**	**True lethal**	**True viable %**	**False viable %**	**MCC**
***Glucose***	iMM904 full	FBA	647	10	32	33	95.29	23.26	0.60
	iMM904 full	linMOMA	644	10	35	33	94.85	23.26	0.58
	iMM904 full	MOMA	644	10	35	33	94.85	23.26	0.58
	iMM904 reduced	FBA	440	9	28	33	94.02	21.43	0.61
	iMM904 reduced	linMOMA	437	9	31	33	93.38	21.43	0.60
	iMM904 reduced	MOMA	437	9	31	33	93.38	21.43	0.60
	iLL672 full	MOMA	433	9	35	33	92.52	21.43	0.57

***Galactose***	iMM904 full	FBA	595	32	36	59	94.29	35.16	0.58
	iMM904 full	linMOMA	595	32	36	59	94.29	35.16	0.58
	iMM904 full	MOMA	595	32	36	59	94.29	35.16	0.58
	iMM904 reduced	FBA	409	12	33	56	92.53	17.65	0.67
	iMM904 reduced	linMOMA	409	12	33	56	92.53	17.65	0.67
	iMM904 reduced	MOMA	409	12	33	56	92.53	17.65	0.67
	iLL672 full	MOMA	411	19	31	49	92.99	27.94	0.61

***Glycerol***	iMM904 full	FBA	596	43	36	47	94.30	47.78	0.48
	iMM904 full	linMOMA	595	44	37	46	94.15	48.89	0.47
	iMM904 full	MOMA	598	44	34	46	94.62	48.89	0.48
	iMM904 reduced	FBA	410	20	34	46	92.34	30.30	0.57
	iMM904 reduced	linMOMA	409	21	35	45	92.12	31.82	0.56
	iMM904 reduced	MOMA	412	21	32	45	92.79	31.82	0.57
	iLL672 full	MOMA	406	20	38	46	91.44	30.30	0.55

***Ethanol***	iMM904 full	FBA	593	45	29	55	95.34	45.00	0.54
	iMM904 full	linMOMA	592	45	30	55	95.18	45.00	0.54
	iMM904 full	MOMA	592	44	30	56	95.18	44.00	0.55
	iMM904 reduced	FBA	408	21	27	54	93.79	28.00	0.64
	iMM904 reduced	linMOMA	407	21	28	54	93.56	28.00	0.63
	iMM904 reduced	MOMA	407	20	28	55	93.56	26.67	0.64
	iLL672 full	MOMA	401	13	34	62	92.18	17.33	0.68

MCC, Matthews correlation coefficient (see Methods). Note that the *i*LL672 predictions were obtained directly from [3] and thus the viability threshold was not optimized using the maximum MCC approach.

#### Inferring perturbed metabolic regions based on EM profiles

The method implemented in this study is shown schematically in Figures 1 and 2 and the steps are described as follows.

### Constraining the *i*MM904 network

Relative levels of quantitative EM data were incorporated into the constraint-based framework as overflow secretion exchange fluxes to simulate the required low-level production of experimentally observed excreted metabolites. The primary objective of this study is to associate relative metabolite levels that are generally measured for metabonomic or biofluid analyses to the quantitative ranges of intracellular reaction fluxes required to produce them. However, without detailed kinetic information or dynamic metabolite measurements available, we approximated EM datasets of relative quantitative metabolite levels to be proportional to the rate in which they are secreted and detected (at a steady state) into the extracellular media. This approach is analogous to approximating uptake rates based on metabolite concentrations from a previous study performing sampling analysis on a cardiomyocyte mitochondrial network to identify differential flux distribution ranges for various environmental (i.e. substrate uptake) conditions [19].

The raw data was normalized by the raw maximum value of the dataset (thus the maximum secretion flux was 1 mmol/hr/gDW) with an assumed error of 10% to set the lower and upper bounds and thus inherently accounting for sampling calculation sensitivity. The *gdh1/GDH2 *strains were flask cultured under minimal glucose media conditions; thus, glucose and oxygen uptake rates were set at 15 and 2 mmol/hr/gDW, respectively, for the *gdh1/GDH2 *strain study. In the anaerobic case the oxygen uptake rate was set to zero, and sterols and fatty acids were provided as *in silico *supplements as described in [35]. For the potassium limitation/ammonium toxicity study the growth rate was set at 0.17 1/h, and the glucose uptake rate was minimized to mimic experimental chemostat cultivation conditions. These input constraints were constant for each perturbation and comparative wild-type condition such that the calculated solution spaces between the conditions differed based only on variations in the output secretion constraints.

### FBA optimization of EM-constrained networks

A modified FBA method with minimization of the 1-norm objective function between two optimal flux distributions was used to determine optimal intracellular fluxes based on the EM-constrained metabolic models. This method determines two optimal flux distributions simultaneously for two differently constrained models (e.g. wild type vs. mutant) – these flux distributions maximize biomass production in each case and the 1-norm distance between the distributions is as small as possible given the two sets of constraints. This approach avoids problems with alternative optimal solutions when comparing two FBA-computed flux distributions by assuming minimal rerouting of flux distibution between a perturbed network and its reference network. Reaction flux changes from the FBA optimization results were determined by computing the relative percentage fold change for each reaction between the mutant and wild-type flux distributions.

### Random sampling of the steady-state solution space

We utilized artificial centering hit-and-run (ACHR) Monte Carlo sampling [19,41] to uniformly sample the metabolic flux solution space defined by the constraints described above. Reactions, and their participating metabolites, found to participate in intracellular loops [42] were discarded from further analysis as these reactions can have arbitrary flux values. The following sections describe the approaches used for the analysis of the different datasets.

#### Sampling approach used in the gdh1/GDH2 study

Due to the overall shape of the metabolic flux solution space, most of the sampled flux distributions resided close to the minimally allowed growth rate (i.e. biomass production) and corresponded to various futile cycles that utilized substrates but did not produce significant biomass. In order to study more physiologically relevant portions of the flux space we restricted the sampling to the part of the solution space where the growth rate was at least 50% of the maximum growth rate for the condition as determined by FBA. This assumes that cellular growth remains an important overall objective by the yeast cells even in batch cultivation conditions, but that the intracellular flux distributions may not correspond to maximum biomass production [43].

To test the sensitivity of the results to the minimum growth rate threshold, separate Monte Carlo samples were created for each minimum threshold ranging from 50% to 100% at 5% increments. We also tested the sensitivity of the results to the relative magnitude of the extracellular metabolite secretion rates by performing the sampling at three different relative levels (0 corresponding to no extracellular metabolite secretion, maximum rate of 0.5 mmol/hr/gDW, and maximum rate of 1.0 mmol/hr/gDW). For each minimum growth rate threshold and extracellular metabolite secretion rate, the ACHR sampler was run for 5 million steps and a flux distribution was stored every 5000 steps. The sensitivity analysis results are presented in Figures S3 and S4 [see Additional File 3], and the results indicate that the reaction *Z*-scores (see below) are not significantly affected by either the portion of the solution space sampled or the exact scaling of secretion rates. The final overall sample used was created by combining the samples for all minimum growth rate thresholds for the highest extracellular metabolite secretion rate (maximum 1 mmol/hr/gDW). This approach allowed biasing the sampling towards physiologically relevant parts of the solution space without imposing the requirement of strictly maximizing a predetermined objective function. The samples obtained with no EM data were used as control samples to filter reporter metabolites/subsystems whose scores were significantly high due to only random differences between sampling runs.

#### Sampling approach used in the potassium limitation/ammonium toxicity study

Since the experimental data used in this study was generated in chemostat conditions, and previous studies have indicated that chemostat flux patterns predicted by FBA are close to the experimentally measured ones [43], we assumed that sampling of the optimal solution space was appropriate for this study. In order to sample a physiologically reasonable range of flux distributions, samples for four different oxygen uptake rates (1, 2, 3, and 4 mmol/hr/gDW with 5 million steps each) were combined in the final analysis.

### Standardized scoring of flux differences between perturbation and control conditions

A *Z*-score based approach was implemented to quantify differences in flux samples between two conditions (Figure 2). First, two flux vectors were chosen randomly, one from each of the two samples to be compared and the difference between the flux vectors was computed. This approach was repeated to create a sample of 10,000 (*n*) flux difference vectors for each pair of conditions considered (e.g. mutant or perturbed environment vs. wild type). Based on this flux difference sample, the sample mean (*μ*_*diff*,*i*_) and standard deviation (*σ*_*diff*,*i*_) between the two conditions was calculated for each reaction *i*. The reaction *Z*-score was calculated as:



which describes the sampled mean difference deviation from a population mean change of zero (i.e. no flux difference between perturbation and wild type). Note that this approach allows accounting for uncertainty in the flux distributions inferred based on the extracellular metabolite secretion constraints. This is in contrast to approaches such as FBA or MoMA that would predict a single flux distribution for each condition and thus potentially overestimate differences between conditions.

The reaction *Z*-scores can then be further used in analysis to identify significantly perturbed regions of the metabolic network based on reporter metabolite [44] or subsystem [30]* Z*-scores. These reporter regions indicate, or "report", dominant perturbation features at the metabolite and pathway levels for a particular condition. The reporter metabolite *Z*-score for any metabolite *j *can be derived from the reaction *Z*-scores of the reactions consuming or producing j (set of reactions denoted as *R*_*j*_) as:



where *N*_*j *_is the number of reactions in *R*_*j *_and m_*met*,*j *_is calculated as



To account and correct for background distribution, the metabolite *Z*-score was normalized by computing *μ*_*met*,*Nj *_and *σ*_*met*,,*Nj *_corresponding to the mean *m*_*met *_and its standard deviation for 1,000 randomly generated reaction sets of size *N*_*j*_. *Z*-scores for subsystems were calculated similarly by considering the set of reactions *R*_*k *_that belongs to each subsystem *k*. Hence, positive metabolite and subsystem scores indicate a significantly perturbed metabolic region relative to other regions, whereas a negative score indicate regions that are not perturbed more significantly than what is expected by random chance. Perturbation subnetworks of reactions and connecting metabolites were visualized using Cytoscape [45].

## Results and discussion

### I. Reconstruction and validation of *i*MM904 network

#### *i*MM904 network content

A previously reconstructed *S. cerevisiae *network, *i*ND750, was used as the basis for the construction of the expanded *i*MM904 network. Prior to its presentation here, the *i*MM904 network content was the basis for a consensus jamboree network that was recently published but has not yet been adapted for FBA calculations [46]. The majority of *i*ND750 content was carried over and further expanded on to construct *i*MM904, which accounts for 904 genes, 1,228 individual metabolites, and 1,412 reactions of which 395 are transport reactions. Both the number of gene-associated reactions and the number of metabolites increased in *i*MM904 compared with the *i*ND750 network. Additional genes and reactions included in the network primarily expanded the lipid, transport, and carbohydrate subsystems. The lipid subsystem includes new genes and reactions involving the degradation of sphingolipids and glycerolipids. Sterol metabolism was also expanded to include the formation and degradation of steryl esters, the storage form of sterols. The majority of the new transport reactions were added to connect network gaps between intracellular compartments to enable the completion of known physiological functions. We also added a number of new secretion pathways based on experimentally observed secreted metabolites [31].

A number of gene-protein-reaction (GPR) relationships were modified to include additional gene products that are required to catalyze a reaction. For example, the protein compounds thioredoxin and ferricytochrome C were explicitly represented as compounds in *i*ND750 reactions, but the genes encoding these proteins were not associated with their corresponding GPRs. Other examples include glycogenin and NADPH cytochrome p450 reductases (CPRs), which are required in the assembly of glycogen and to sustain catalytic activity in cytochromes p450, respectively. These additional proteins were included in *i*MM904 as part of protein complexes to provide a more complete representation of the genes and their corresponding products necessary for a catalytic activity to occur.

Major modifications to existing reactions were in cofactor biosynthesis, namely in quinone, beta-alanine, and riboflavin biosynthetic pathways. Reactions from previous *S. cerevisiae *networks associated with quinone, beta-alanine, and riboflavin biosynthetic pathways were essentially inferred from known reaction mechanisms based on reactions in previous network reconstructions of *E. coli *[2,47]. These pathways were manually reviewed based on current literature and subsequently replaced by reactions and metabolites specific to yeast. Additional changes in other subsystems were also made, such as changes to the compartmental location of a gene and its corresponding reaction(s), changes in reaction reversibility and cofactor specificity, and the elucidation of particular transport mechanisms. A comprehensive listing of *i*MM904 network contents as well as a detailed list of changes between *i*ND750 and *i*MM904 is included [see Additional file 1].

#### Predicting deletion growth phenotypes

The updated genome-scale *i*MM904 metabolic network was validated by comparing *in silico *single-gene deletion predictions to *in vivo *results from a previous study used to analyze another *S. cerevisiae *metabolic model, *i*LL672 [3]. This network was constructed based on the *i*FF708 network [22], which was also the starting point for reconstructing the *i*ND750 network [2]. The experimental data used to validate the *i*LL672 model consisted of 3,360 single-gene knockout strain phenotypes evaluated under minimal media growth conditions with glucose, galactose, glycerol, and ethanol as sole carbon sources. Growth phenotypes for the *i*MM904 network were predicted using FBA [32-34], MoMA [35], and linear MoMA methods as described in Methods and subsequently compared to the experimental data (Table 1). Each deleted gene growth prediction comparison was classified as true lethal, true viable, false lethal, or false viable. The growth rate threshold for considering a prediction viable was chosen for each condition and method separately to optimize the tradeoff between true viable and false viable predictions (maximum Matthews correlation coefficient, see Methods).

Since *i*MM904 has 212 more genes than *i*LL672 with experimental data, we also present results for the subset of *i*MM904 predictions with genes included in *i*LL672 (reduced *i*MM904 set). When the same gene sets are compared, *i*MM904 improves gene lethality predictions under glucose, galactose, and glycerol conditions over *i*LL672 somewhat, but is less accurate at predicting growth phenotypes under the ethanol condition. It should be noted that the *i*LL672 predictions were obtained directly from [3] and thus the growth rate threshold was not optimized similarly to *i*MM904 predictions. Overall, when viability cutoff is chosen as indicated above for each method separately, the three prediction methods (FBA, MOMA, and linear MOMA) perform similarly.

While the full gene complement in *i*MM904 greatly increased the number of true viable predictions, the full model also made significantly more false viable predictions compared with reduced *i*MM904 and *i*LL672 predictions. However, it is important to note that 143 reactions involved in dead-end biosynthetic pathways were actually removed from *i*FF708 to build the *i*LL672 reconstruction [3]. These dead-ends are considered "knowledge gaps" in pathways that have not been fully characterized and, as a result, lead to false viable predictions when determining gene essentiality if the pathway is in fact required for growth under a certain condition [2,26]. As more of these pathways are elucidated and included in the model to fill in existing network gaps, we can expect false viable prediction rates to consequently decrease. Thus, while a larger network has a temporarily reduced capacity to accurately predict gene deletion phenotypes, it captures a more complete picture of currently known metabolic functions and provides a framework for network expansion as new pathways are elucidated [48].

### II. Inferring intracellular perturbation states from metabolic profiles

#### Aerobic and anaerobic gdh1/GDH2 mutant behavior

The *gdh1/GDH2 *mutant strain was previously developed [49,50] in order to lower NADPH consumption in ammonia assimilation, which would in turn favor the NADPH-dependent fermentation of xylose. In this strain, the NADPH-dependent glutamate dehydrogenase, *Gdh1*, was deleted and the NADH-dependent form of the enzyme, *Gdh2*, was overexpressed. The net effect is to allow efficient assimilation of ammonia into glutamate using NADH instead of NADPH as a cofactor. While growth characteristics remained unaffected, relative quantities of secreted metabolites differed between the wild-type and mutant strain under aerobic and anaerobic conditions.

We analyzed EM data for the *gdh1/GDH2 *and wild-type strains reported in [31] under aerobic and anaerobic conditions separately using both FBA optimization and sampling-based approaches as described in Methods. 43 measured extracellular and intracellular metabolites from the original dataset [31], primarily of central carbon and amino acid metabolism, were explicitly represented in the *i*MM904 network [see Additional file 4]. Extracellular metabolite levels were used to formulate secretion constraints and differential intracellular metabolites were used to compare and validate the intracellular flux predictions. Perturbed reactions from the FBA results were determined by calculating relative flux changes, and reaction *Z*-scores were calculated from the sampling analysis to quantify flux changes between the mutant and wild-type strains, with Z_reaction _> 1.96 corresponding to a two-tailed *p*-value < 0.05 and considered to be significantly perturbed [see Additional file 4].

To validate the predicted results, reaction flux changes from both FBA and sampling methods were compared to differential intracellular metabolite level data measured from the same study. Intracellular metabolites involved in highly perturbed reactions (i.e. reactants and products) predicted from FBA and sampling analyses were identified and compared to metabolites that were experimentally identified as significantly changed (*p *< 0.05) between mutant and wild-type. Statistical measures of recall, accuracy, and precision were calculated and represent the predictive sensitivity, exactness, and reproducibility respectively. From the sampling analysis, a considerably larger number of significantly perturbed reactions are predicted in the anaerobic case (505 reactions, or 70.7% of active reactions) than in aerobic (394 reactions, or 49.8% of active reactions). The top percentile of FBA flux changes equivalent to the percentage of significantly perturbed sampling reactions were compared to the intracellular data. Results from both analyses are summarized in Table 2. Sampling predictions were considerably higher in recall than FBA predictions for both conditions, with respective ranges of 0.83–1 compared to 0.48–0.96. Accuracy was also higher in sampling predictions; however, precision was slightly better in the FBA predictions as expected due to the smaller number of predicted changes. Overall, the sampling predictions of perturbed intracellular metabolites are strongly consistent with the experimental data and significantly outperforms that of FBA optimization predictions in accurately predicting differential metabolites involved in perturbed intracellular fluxes.

**Table 2:** Statistical comparison of the differential intracellular metabolite data set (*p *< 0.05) with metabolites involved in perturbed reactions predicted by FBA optimization and sampling analyses for aerobic and anaerobic *gdh1/GDH2 *mutant.

	**Aerobic**		**Anaerobic**		**Overall**	
	*FBA*	*Sampling*	*FBA*	*Sampling*	*FBA*	*Sampling*
*Recall*	0.48	0.83	0.96	1.00	0.71	0.91
*Accuracy*	0.55	0.62	0.64	0.64	0.60	0.63
*Precision*	0.78	0.69	0.64	0.63	0.68	0.66

Overall statistics indicate combined results of both conditions.

**Figure 3:**
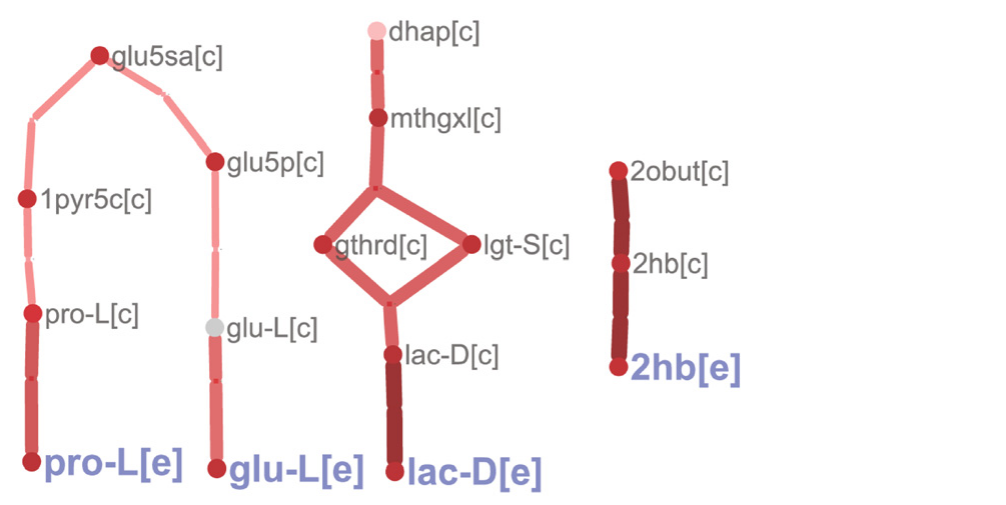
**Perturbation reaction subnetwork of *gdh1/GDH2 *mutant under aerobic conditions**. The network illustrates a simplified subset of highly perturbed reactions connected to aerobically-secreted metabolites predicted from the sampling analysis of the *gdh1/GDH2 *mutant strain. The major secreted metabolites (glutamate, proline, D-lactate, and 2-hydroxybuturate) were also detected in the anaerobic condition. Metabolite abbreviations are found in Additional file 1.

**Figure 4:**
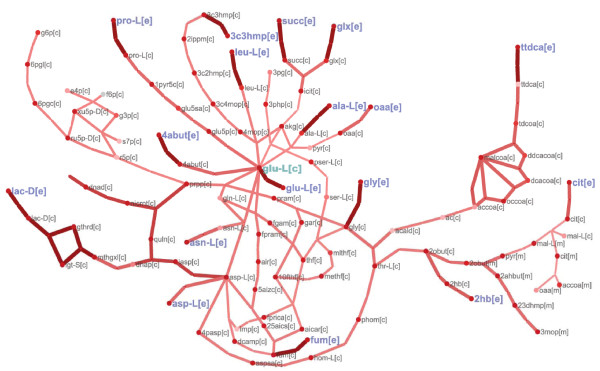
**Perturbation reaction subnetwork of *gdh1/GDH2 *mutant under anaerobic conditions**. Subnetwork illustrates the highly perturbed anaerobic reactions of similar *Z*_reaction _magnitude to the reactions in Figure 3. A significantly larger number of reactions indicates mutant metabolic effects are more widespread in the anaerobic environment. The network shows that perturbed pathways converge on glutamate, the main site in which the *gdh1/GDH2 *modification was introduced, which suggests that the direct genetic perturbation effects are amplified under this environment. Metabolite abbreviations are found in Additional file 1.

Perturbation subnetworks can be drawn to visualize predicted significantly perturbed intracellular reactions and illustrate their connection to the observed secreted metabolites in the aerobic and anaerobic *gdh1/GDH2 *mutants. Figure 3 shows an example of a simplified aerobic perturbation subnetwork consisting primarily of proximal pathways connected directly to a subset of major secreted metabolites (glutamate, proline, D-lactate, and 2-hydroxybuturate). Figure 4 displays anaerobic reactions with Z-scores of similar magnitude to the perturbed reactions in Figure 3. The same subset of metabolites is also present in the larger anaerobic perturbation network and indicates that the NADPH/NADH balance perturbation induced by the *gdh1/GDH2 *manipulation has widespread effects beyond just altering glutamate metabolism anaerobically. Interestingly, it is clear that the majority of the secreted metabolite pathways involve connected perturbed reactions that broadly converge on glutamate. Note that Figures 3 and 4 only show the subnetworks that consisted of two or more connected reactions – for a number of secreted metabolites no contiguous perturbed pathway could be identified by the sampling approach. This indicates that the secreted metabolite pattern alone is not sufficient to determine which specific production and secretion pathways are used by the cell for these metabolites.

To further highlight metabolic regions that have been systemically affected by the *gdh1/GDH2 *modification, reporter metabolite and subsystem methods [30] were used to summarize reaction scores around specific metabolites and in specific metabolic subsystems. The top ten significant scores for metabolites/subsystems associated with more than three reactions are summarized in Tables 3 (aerobic) and 4 (anaerobic), with Z > 1.64 corresponding to *p *< 0.05 for a one-tailed distribution. Full data for all reactions, reporter metabolites, and reporter subsystems is included [see Additional file 4].

**Table 3:** List of the top ten significant reporter metabolite and subsystem scores for the *gdh1/GDH2 *vs. wild type comparison in aerobic conditions.

**Reporter metabolite**	**Z-score**	No of reactions*
L-proline [c]	2.71	4
Carbon dioxide [m]	2.51	15
Proton [m]	2.19	51
Glyceraldehyde 3-phosphate [c]	1.93	7
Ubiquinone-6 [m]	1.82	5
Ubiquinol-6 [m]	1.82	5
Ribulose-5-phosphate [c]	1.80	4
Uracil [c]	1.74	4
L-homoserine [c]	1.72	4
Alpha-ketoglutarate [m]	1.71	8

**Reporter subsystem**	**Z-score**	**No of reactions**

Citric Acid Cycle	4.58	7
Pentose Phosphate Pathway	3.29	12
Glycine and Serine Metabolism	2.69	17
Alanine and Aspartate Metabolism	2.65	6
Oxidative Phosphorylation	1.79	8
Thiamine Metabolism	1.54	8
Arginine and Proline Metabolism	1.44	20
Other Amino Acid Metabolism	1.28	5
Glycolysis/Gluconeogenesis	0.58	14
Anaplerotic reactions	0.19	9

*Number of reactions categorized in a subsystem or found to be neighboring each metabolite

**Table 4:** List of top ten significant reporter metabolite and subsystem scores for the *gdh1/GDH2 *vs. wild type comparison in anaerobic conditions.

**Reporter metabolite**	**Z-score**	**No of reactions**
Glutamate [c]	4.52	35
Aspartate [c]	3.21	11
Alpha-ketoglutarate [c]	2.66	17
Glycine [c]	2.65	7
Pyruvate [m]	2.56	7
Ribulose-5-phosphate [c]	2.43	4
Threonine [c]	2.28	6
10-formyltetrahydrofolate [c]	2.27	5
Fumarate [c]	2.27	5
L-proline [c]	2.04	4

**Reporter subsystem**	**Z-score**	**No of reactions**

Valine, Leucine, and Isoleucine Metabolism	3.97	15
Tyrosine, Tryptophan, and Phenylalanine Metabolism	3.39	23
Pentose Phosphate Pathway	3.29	11
Purine and Pyrimidine Biosynthesis	3.08	40
Arginine and Proline Metabolism	2.96	19
Threonine and Lysine Metabolism	2.74	14
NAD Biosynthesis	2.66	7
Alanine and Aspartate Metabolism	2.65	6
Histidine Metabolism	2.24	10
Cysteine Metabolism	1.85	10

Perturbations under aerobic conditions largely consisted of pathways involved in mediating the NADH and NADPH balance. Among the highest scoring aerobic subsystems are TCA cycle and pentose phosphate pathway – key pathways directly involved in the generation of NADH and NADPH. Reporter metabolites involved in these subsystems – glyceraldehyde-3-phosphate, ribulose-5-phosphate, and alpha-ketoglutarate – were also identified. These results are consistent with flux and enzyme activity measurements of the *gdh1/GDH2 *strain under aerobic conditions [32], which reported significant reduction in the pentose phosphate pathway flux with concomitant changes in other central metabolic pathways. Levels of several TCA cycle intermediates (e.g. fumarate, succinate, malate) were also elevated in the *gdh1/GDH2 *mutant according to the differential intracellular metabolite data. Altered energy metabolism, as indicated by reporter metabolites (i.e. ubiquinone-6, ubiquinol-6, mitochondrial proton) and subsystem (oxidative phosphorylation), is certainly feasible as NADH is a primary reducing agent for ATP production. Pentose phosphate pathway and NAD biosynthesis also appears among the most perturbed anaerobic subsystems, further suggesting perturbed cofactor balance as a common, dominant effect under both conditions.

Glutamate dehydrogenase is a critical enzyme of amino acid biosynthesis as it acts as the entry point for ammonium assimilation via glutamate. Consequently, metabolic subsystems involved in amino acid biosynthesis were broadly perturbed as a result of the *gdh1/GDH2 *modification in both aerobic and anaerobic conditions. For example, the proline biosynthesis pathway that uses glutamate as a precursor was significantly perturbed in both conditions, as supported by significantly changed intracellular and extracellular levels. There were differences, however, in that more amino acid related subsystems were significantly affected in the anaerobic case (Table 4), further highlighting that altered ammonium assimilation in the mutant has a more widespread effect under anaerobic conditions. This effect is especially pronounced for threonine and nucleotide metabolism, which were predicted to be significantly perturbed only in anaerobic conditions. Intracellular threonine levels were amongst the most significantly reduced relative to other differential intracellular metabolites in the anaerobically grown *gdh1/GDH2 *strain (see [31] and Additional file 4), and the relationship between threonine and nucleotide biosynthesis is further supported by threonine's recently discovered role as a key precursor in yeast nucleotide biosynthesis [51]. Other key anaerobic reporter metabolites are glycine and 10-formyltetrahydrofolate, both of which are involved in the cytosolic folate cycle (one-carbon metabolism). Folate is intimately linked to biosynthetic pathways of glycine (with threonine as its precursor) and purines by mediating one-carbon reaction transfers necessary in their metabolism and is a key cofactor in cellular growth [52]. Thus, the anaerobic perturbations identified in the analysis emphasize the close relationship between threonine, folate, and nucleotide metabolic pathways as well as their potential connection to perturbed ammonium assimilation processes. Interestingly, this association has been previously demonstrated at the transcriptional level as yeast ammonium assimilation (via glutamine synthesis) was found to be co-regulated with genes involved in glycine, folate, and purine synthesis [53].

In summary, the overall differences in predicted *gdh1/GDH2 *mutant behavior under aerobic and anaerobic conditions show that changes in flux states directly related to modified ammonium assimilation pathway are amplified anaerobically whereas the indirect effects through NADH/NADPH balance are more significant aerobically. Perturbed metabolic regions under aerobic conditions were predominantly in central metabolic pathways involved in responding to the changed NADH/NADPH demand and did not necessarily emphasize that glutamate dehydrogenase was the site of the genetic modification. The majority of affected anaerobic pathways were involved directly in modified ammonium assimilation as evidenced by 1) significantly perturbed amino acid subsystems, 2) a broad perturbation subnetwork converging on glutamate (Figure 4), and 3) glutamate as the most significant reporter metabolite (Table 4).

#### Potassium-limited and excess ammonium environments

A recent study reported that potassium limitation resulted in significant growth retardation effect in yeast due to excess ammonium uptake when ammonium was provided as the sole nitrogen source [33]. The proposed mechanism for this effect was that ammonium could to be freely transported through potassium channels when potassium concentrations were low in the media environment, thereby resulting in excess ammonium uptake [33]. As a result, yeast incurred a significant metabolic cost in assimilating ammonia to glutamate and secreting significant amounts of glutamate and other amino acids in potassium-limited conditions as a means to detoxify the excess ammonium. A similar effect was observed when yeast was grown with no potassium limitation, but with excess ammonia in the environment. While the observed effect of both environments (low potassium or excess ammonia) was similar, quantitatively unique amino acid secretion profiles suggested that internal metabolic states in these conditions are potentially different.

**Figure 5:**
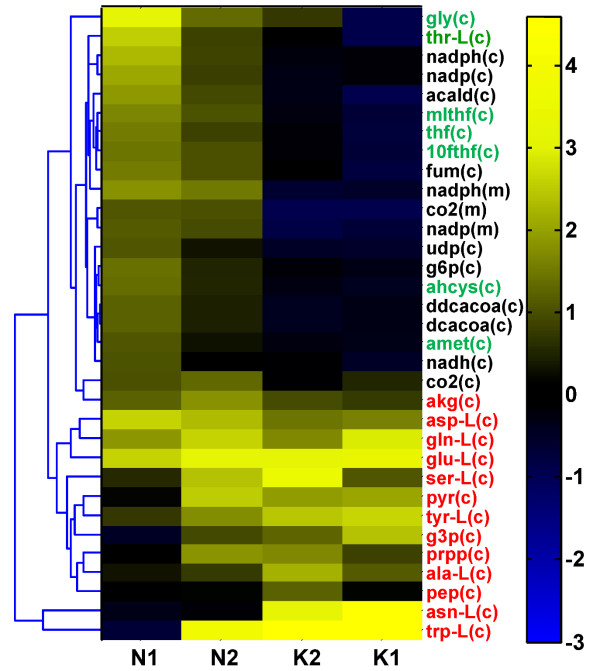
**Clustergram of top reporter metabolites (i.e. in yellow) in ammonium-toxic and potassium-limited conditions**. Amino acid perturbation patterns (shown in red) were shown to be consistently scored across conditions, indicating that potassium-limited environments K1 (lowest concentration) and K2 (low concentration) elicited a similar ammonium detoxification response as ammonium-toxic environments N1 (high concentration) and N2 (highest concentration). Metabolites associated with folate metabolism (highlighted in green) are also highly perturbed in ammonium-toxic conditions. Metabolite abbreviations are found in Additional file 1.

In order to elucidate the differences in internal metabolic states, we utilized the *i*MM904 model and the EM profile analysis method to analyze amino acid secretion profiles for a range of low potassium and high ammonia conditions reported in [33]. As before, we utilized amino acid secretion patterns as constraints to the *i*MM904 model, sampled the allowable solution space, computed reaction *Z*-scores for changes from a reference condition (normal potassium and ammonia), and finally summarized the resulting changes using reporter metabolites. Figure 5 shows a clustering of the most significant reporter metabolites (Z ≥ 1.96 in any of the four conditions studied) obtained from this analysis across the four conditions studied. Interestingly, the potassium-limited environment perturbed only a subset of the significant reporter metabolites identified in the high ammonia environments. Both low potassium environments shared a consistent pattern of highly perturbed amino acids and related precursor biosynthesis metabolites (e.g. pyruvate, PRPP, alpha-ketoglutarate) with high ammonium environments. The amino acid perturbation pattern (indicated by red labels in Figure 5) was present in the ammonium-toxic environments, although the pattern was slightly weaker for the lower ammonium concentration. Nevertheless, the results clearly indicate that a similar ammonium detoxifying mechanism that primarily perturbs pathways directly related to amino acid metabolism exists under both types of media conditions.

In addition to perturbed amino acids, a secondary effect notably appears at high ammonia levels in which metabolic regions related to folate metabolism are significantly affected. As highlighted in green in Figure 3, we predicted significantly perturbed key metabolites involved in the cytosolic folate cycle. These include tetrahydrofolate derivatives and other metabolites connected to the folate pathway, namely glycine and the methionine-derived methylation cofactors S-adenosylmethionine and S-adenosylhomocysteine. Additionally, threonine was identified to be a key perturbed metabolite in excess ammonium conditions. These results further illustrate the close connection between threonine biosynthesis, folate metabolism involving glycine derived from its threonine precursor, and nucleotide biosynthesis [51] that was discussed in conjunction with the *gdh1/GDH2 *strain data. Taken together with the anaerobic *gdh1/GDH2 *data, the results consistently suggest highly perturbed threonine and folate metabolism when amino acid-related pathways are broadly affected.

In both ammonium-toxic and potassium-limited environments, impaired cellular growth was observed, which can be attributed to high energetic costs of increased ammonium assimilation to synthesize and excrete amino acids. However, under high ammonium environments, reporter metabolites related to threonine and folate metabolism indicated that their perturbation, and thus purine supply, may be an additional factor in decreasing cellular viability as there is a direct relationship between intracellular folate levels and growth rate [54]. Based on these results, we concluded that while potassium-limited growth in yeast indeed shares physiological features with growth in ammonium excess, its effects are not as detrimental as actual ammonium excess. The effects on proximal amino acid metabolic pathways are similar in both environments as indicated by the secretion of the majority of amino acids. However, when our method was applied to analyze the physiological basis behind differences in secretion profiles between low potassium and high ammonium conditions, ammonium excess was predicted to likely disrupt physiological ammonium assimilation processes, which in turn potentially impacts folate metabolism and associated cellular growth.

## Conclusion

The method presented in this study presents an approach to connecting intracellular flux states to metabolites that are excreted under various physiological conditions. We showed that well-curated genome-scale metabolic networks can be used to integrate and analyze quantitative EM data by systematically identifying altered intracellular pathways related to measured changes in the extracellular metabolome. We were able to identify statistically significant metabolic regions that were altered as a result of genetic (*gdh1/GD2 *mutant) and environmental (excess ammonium and limited potassium) perturbations, and the predicted intracellular metabolic changes were consistent with previously published experimental data including measurements of intracellular metabolite levels and metabolic fluxes. Our reanalysis of previously published EM data on ammonium assimilation-related genetic and environmental perturbations also resulted in testable hypotheses about the role of threonine and folate pathways in mediating broad responses to changes in ammonium utilization. These studies also demonstrated that the sampling-based method can be readily applied when only partial secreted metabolite profiles (e.g. only amino acids) are available.

With the emergence of metabolite biofluid biomarkers as a diagnostic tool in human disease [55,56] and the availability of genome-scale human metabolic networks [1], extensions of the present method would allow identifying potential pathway changes linked to these biomarkers. Employing such a method for studying yeast metabolism was possible as the metabolomic data was measured under controllable environmental conditions where the inputs and outputs of the system were defined. Measured metabolite biomarkers in a clinical setting, however, is far from a controlled environment with significant variations in genetic, nutritional, and environmental factors between different patients. While there are certainly limitations for clinical applications, the method introduced here is a progressive step towards applying genome-scale metabolic networks towards analyzing biofluid metabolome data as it 1) avoids the need to only study optimal metabolic states based on a predetermined objective function, 2) allows dealing with noisy experimental data through the sampling approach, and 3) enables analysis even with limited identification of metabolites in the data. The ability to establish potential connections between extracellular markers and intracellular pathways would be valuable in delineating the genetic and environmental factors associated with a particular disease.

## Authors' contributions

Conceived and designed the experiments: MLM MJH BOP. Performed experiments: MLM MJH. Analyzed the data: MLM MJH. Wrote the paper: MLM MJH BOP. All authors have read and approved the final manuscript.

## Supplementary Material

Additional file 1 **iMM904 network content.** The data provided represent the content description of the *i*MM904 metabolic network and detailed information on the expanded content. Click here for file

Additional file 2 **iMM904 model files.** The data provided are the model text files of the *i*MM904 metabolic network that is compatible with the available COBRA Toolbox [13]. The model structure can be loaded into Matlab using the 'SimPhenyPlus' format with GPR and compound information. Click here for file

Additional file 3 **Supplemental figures. **The file provides the supplemental figures and descriptions of S1, S2, S3, and S4. Click here for file

Additional file 4 **Gdh mutant aerobic and anaerobic analysis results. **The data provided are the full results for the exometabolomic analysis of aerobic and anerobic *gdh1/GDH2 *mutant. Click here for file
