# Supplementary material for: Connecting extracellular metabolomic measurements to intracellular flux states in yeast
Source: BMC Syst Biol. 2009 Mar 25;3:37. doi: 10.1186/1752-0509-3-37 (PMC2679711; doi:10.1186/1752-0509-3-37)

Additional File S3.

Supplemental Figures

Figure S1.
Aerobic and anaerobic glucose uptake rates at different dilution rates.
Measured experimental and *i*MM904 values were plotted and shown to be comparably similar under both conditions.

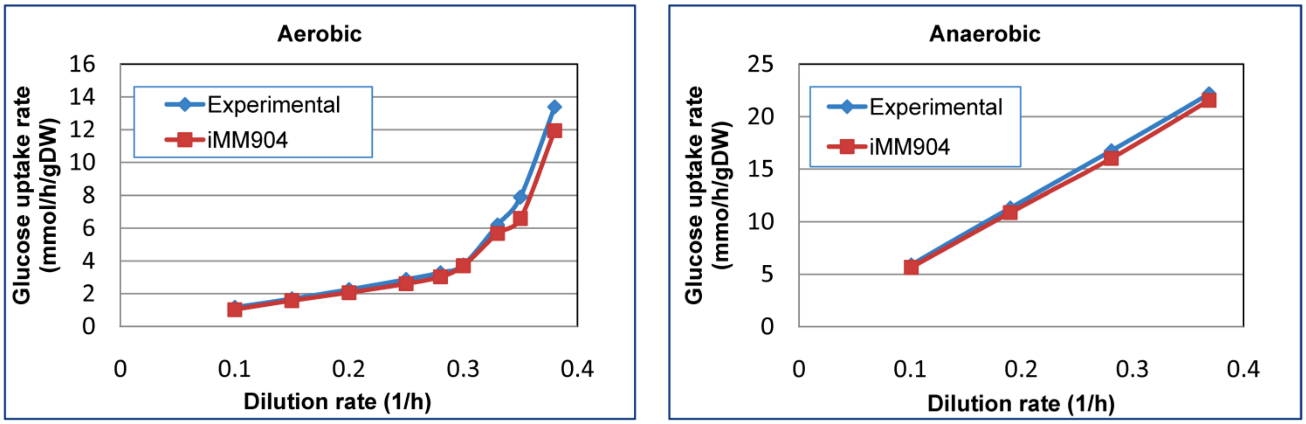

Figure S2.
ROC curve plots of *i*MM904 and *i*LL672 predictions using different analysis methods.

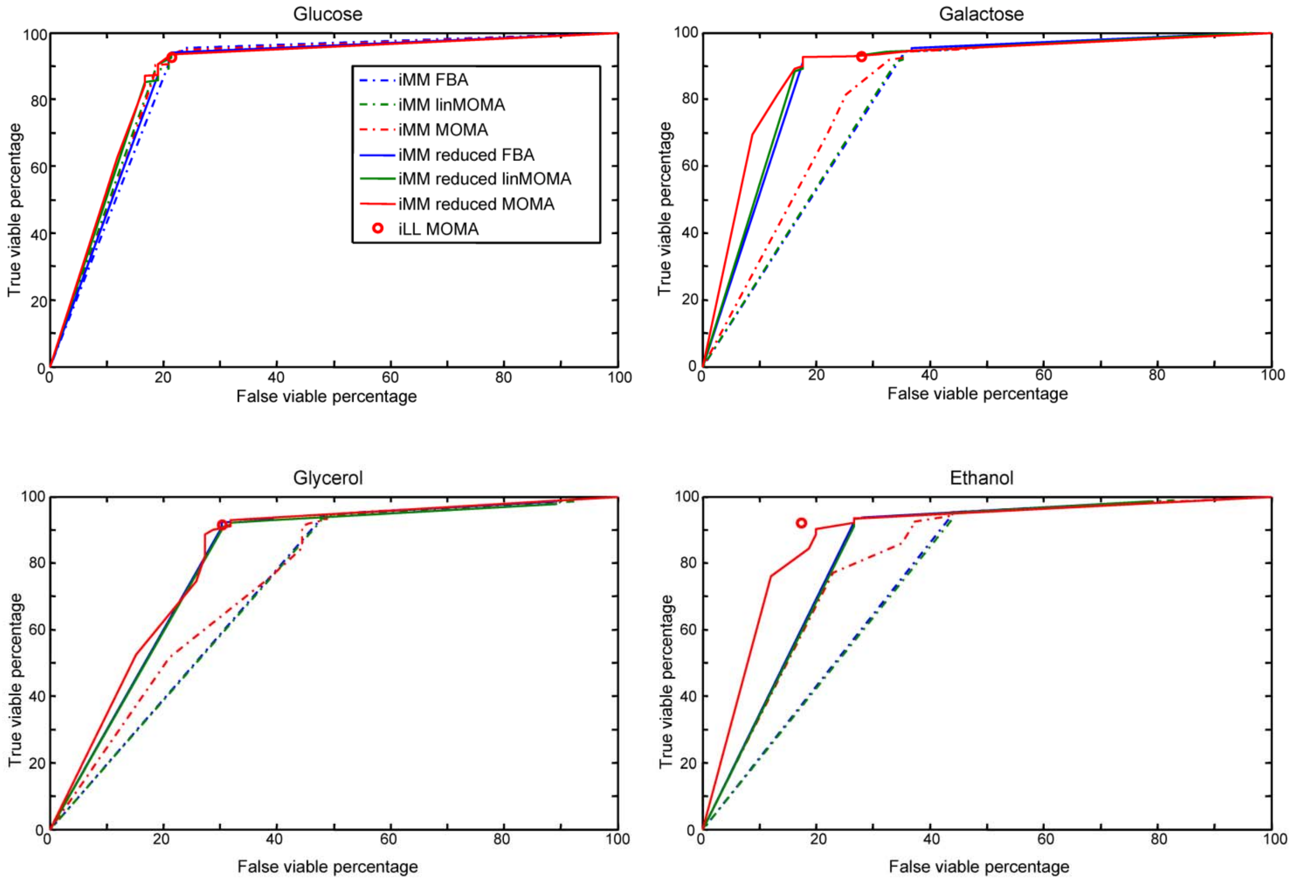

Figure S3.

Sensitivity analysis for reaction *Z*-scores with respect to magnitude (f) of secretion fluxes. Scatterplots indicate that the weighting magnitude of the extracellular metabolite secretion fluxes (i.e.,between f=0.5 and f=1) does not generally affect reaction Z-scores calculations. While reaction Z-scores are sensitive to the addition of secretion flux constraints, the magnitude of the constraints does not significantly affect the scores.

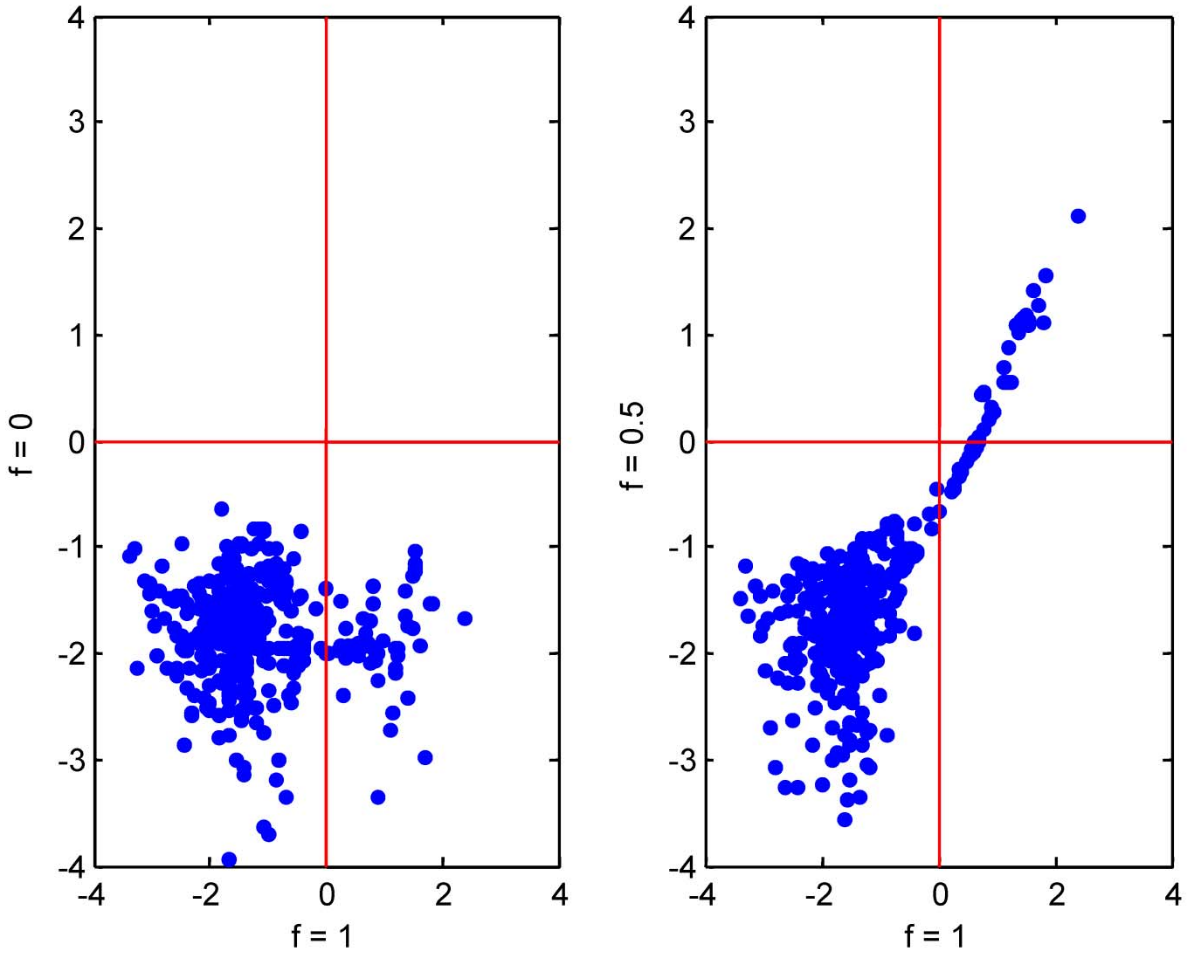

Figure S4.
Sensitivity analysis of reaction *Z*-scores with respect to the percentage of the solution space sampled.
X-axis in each plot shows the *Z*-scores obtained by sampling the upper 50% of the solution space relative to the maximum biomass production rate. (A) Y-axis: *Z*-scores obtained by sampling the range between 50-75% of maximum biomass production rate. (B) Y-axis: *Z*-scores obtained by sampling the upper 25% of the solution space. Reaction Z-scores are independent of either portion (i.e. lower 25% and upper 25%) of the solution space that was sampled between 50-100%; thus, Z-score results remain consistent across the sampled solution space irrespective of the biomass rate constraints.

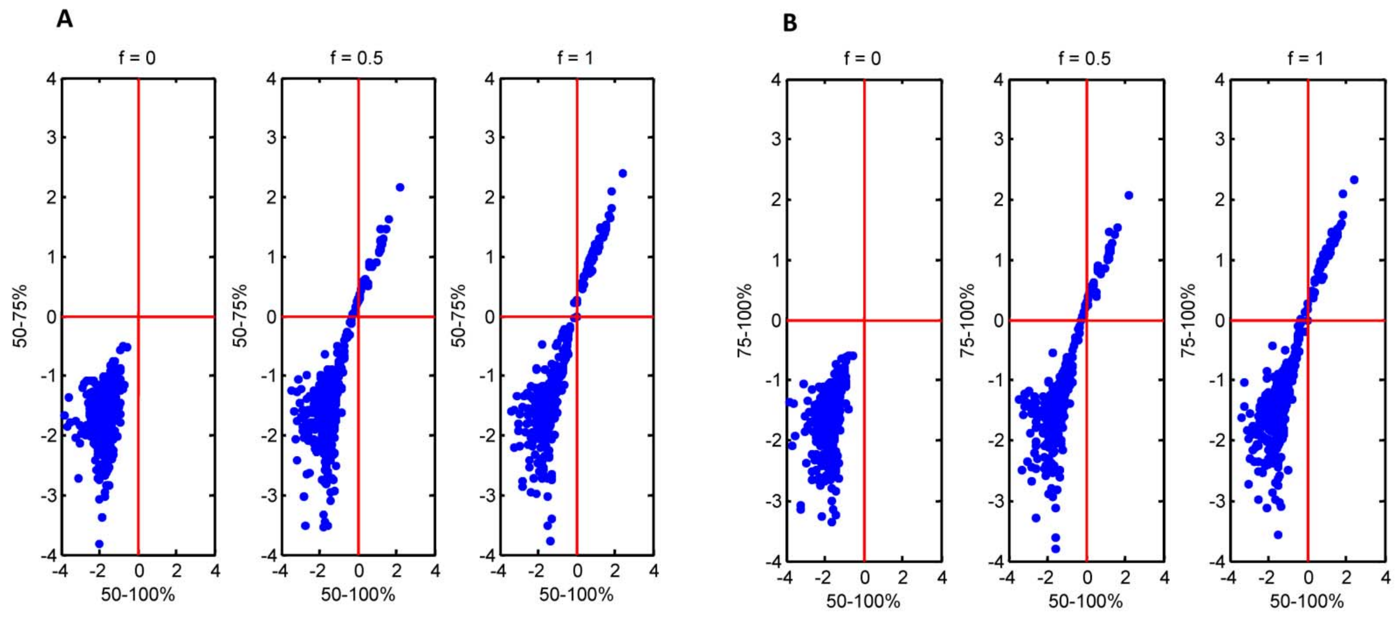

Supplement: Additional file 3 — Supplemental figures. The file provides the supplemental figures and descriptions of S1, S2, S3, and S4. [file 1752-0509-3-37-S3.pdf]
